# Update on transfusion-related acute lung injury: an overview of its pathogenesis and management

**DOI:** 10.3389/fimmu.2023.1175387

**Published:** 2023-05-12

**Authors:** Yunhong Yu, Zhengqiu Lian

**Affiliations:** Department of Blood Transfusion, The Third People’s Hospital of Chengdu, Affiliated Hospital of Southwest Jiaotong University, Chengdu, China

**Keywords:** transfusion-related acute lung injury (TRALI), pathogenesis, priming step, pulmonary reaction, effector phase, management

## Abstract

Transfusion-related acute lung injury (TRALI) is a severe adverse event and a leading cause of transfusion-associated death. Its poor associated prognosis is due, in large part, to the current dearth of effective therapeutic strategies. Hence, an urgent need exists for effective management strategies for the prevention and treatment of associated lung edema. Recently, various preclinical and clinical studies have advanced the current knowledge regarding TRALI pathogenesis. In fact, the application of this knowledge to patient management has successfully decreased TRALI-associated morbidity. This article reviews the most relevant data and recent progress related to TRALI pathogenesis. Based on the existing two-hit theory, a novel three-step pathogenesis model composed of a priming step, pulmonary reaction, and effector phase is postulated to explain the process of TRALI. TRALI pathogenesis stage-specific management strategies based on clinical studies and preclinical models are summarized with an explication of their models of prevention and experimental drugs. The primary aim of this review is to provide useful insights regarding the underlying pathogenesis of TRALI to inform the development of preventive or therapeutic alternatives.

## 1 Introduction

Transfusion of blood components is a widely employed life-saving treatment in clinical settings; however, it can result in potentially life-threatening adverse reactions (1). For example, transfusion-related acute lung injury (TRALI) is a rare but severe adverse event that occurs during—or within—6 hours of blood transfusion and is characterized by hypoxia and non-cardiogenic pulmonary edema, known as respiratory distress (2). In 2019, a new diagnostic criteria was established using the Delphi approach, effectively subdividing TRALI into Type I and II, which occur in the absence or presence of acute respiratory distress syndrome (ARDS) risk factors, respectively (3). Although TRALI represents a main cause of transfusion-associated mortality in developed countries (4), it is likely underdiagnosed, particularly in intensive care patients, owing to the synergetic action of multiple factors (5, 6). Although estimates of TRALI mortality vary markedly between 5% and 25% (7, 8), they can reach 47% in intensive care and surgical patients (9). Even so, specific therapeutics remain unavailable.

When TRALI was first reported in 1957, it was thought to be related to the passive transfer of high-titer leucoagglutinins (10). It was not until 1983 that TRALI was formally identified as a distinctive clinical syndrome caused by transfused anti-human leukocyte antigen (HLA) antibodies (11). However, in addition to the transfer of anti-HLA antibodies, anti-human neutrophil antigen (HNA) antibodies also contribute to TRALI induction. In fact, antibody-dependent mechanisms are estimated to be responsible for 50–80% of TRALI cases (12); however, because of the inaccuracy of current laboratory testing techniques, their contribution has likely been underestimated (13). Moreover, TRALI cases that meet the diagnostic criteria have been reported in patients who received transfused products without antibodies (14). More specifically, approximately 10–15% of TRALI cases are associated with biological response modifiers (BRMs), such as soluble CD40 ligand (sCD40L) or lyso-phosphatidylcholines (lyso-PCs), which originate from the storage of blood components. Thus, non-antibody-mediated TRALI has also been proposed. Although significant progress has been made in the field, the complex pathogenesis of TRALI has not yet been fully characterized.

In this review, we highlight the recent advances in the field regarding TRALI pathogenesis and management based on clinical studies and preclinical models. The findings of these studies provide a clearer understanding of the underlying pathogenesis, which will assist in developing effective prevention or therapeutic strategies.

## 2 Pathogenesis

**Figure 1:**
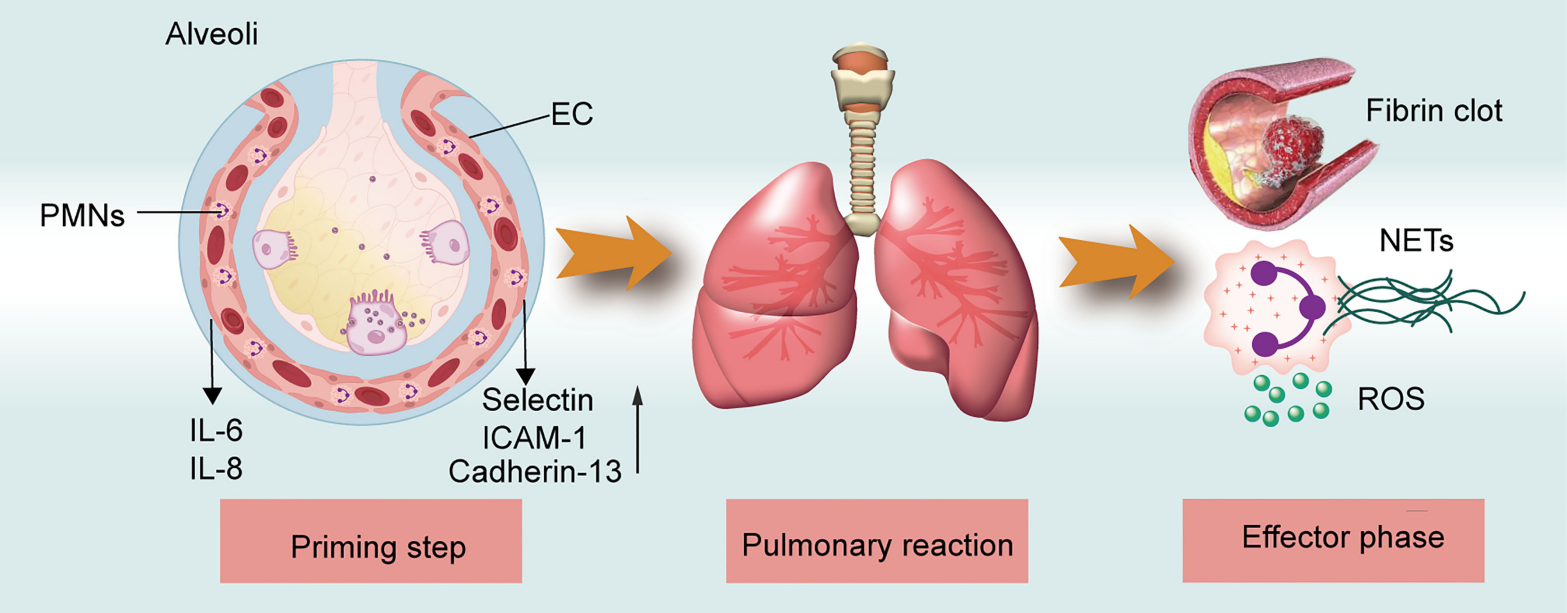
TRALI pathogenesis model. The three-step of TRALI pathogenesis: prime step, pulmonary reaction, and effector step. The pathogenesis of each step is described.

The pathogenesis of TRALI has been described as a two-hit theory, in which recipient predisposition, together with the presence of detrimental factors in blood components, play significant roles (15). That is, the first hit focusing on recipient predisposition, in which pulmonary endothelial cells (ECs) are activated and polymorphonuclear neutrophils (PMNs) are primed. The second hit comprises mediators in transfused stored units, which trigger the primed PMNs and other cells including ECs,monocytes, macrophages, and platelets to release pathogenetic factors and induce coagulopathy, ultimately resulting in fluid intrapulmonary infiltration. The second hit can be classified into antibody-dependent and -independent TRALI based on the detection of differential pathogenic mediators in the blood components. While the two-hit hypothesis clearly explains TRALI pathogenic factors, the disease pathogenesis can be further characterized as three overlapping phases (**Figure 1**), i.e., the priming step, pulmonary reaction, and effector phase. During the priming step, recipient-related risk factors mainly lead to endothelial activation and PMN priming via the EC-PMN interaction. Subsequently, in the pulmonary reaction, antibodies or BRMs bind to target cells, such as ECs, PMNs, or mononuclear cells, thereby inducing a host response. In the final effector phase, the pulmonary vascular endothelial barrier may become damaged by the release of neutrophil extracellular traps (NETs) and reactive oxygen species (ROS); the resulting coagulopathy might aggravate lung injury. The diverse phase characteristics of the three-step pathogenesis model will be described in detail to provide a better understanding of TRALI pathogenesis.

### 2.1 Priming step

As early as 1997, the results of a retrospective study in combination with an *in vivo* rat lung model suggested that stronger PMN-priming activity occurred in patients with TRALI and underlying clinical conditions than in those not diagnosed with TRALI (16). A prospective study revealed that patients diagnosed with hematologic malignance or cardiac disease are at increased risk for developing TRALI (15). Subsequent studies have identified myriad complications that function as first hits, including hypertension, end-stage liver disease, chronic alcohol abuse, surgery, mechanical ventilation, trauma, massive transfusion, and systemic inflammation (13, 17). Based on retrospective cohort studies, pulmonary diseases, including pulmonary fibrosis, interstitial lung disease, and tobacco abuse, are identified as potential high-risk factors for TRALI occurrence (18, 19). Moreover, critical patients with these underlying diseases conveyed a serious first hit, requiring only a minor second hit to trigger TRALI (20). Patients with TRALI in an intensive care unit (ICU) exhibited 70% mortality compared with typical 5–25% rates (20, 21). Thus, underlying clinical events play a pivotal pathogenic role in TRALI onset; screening these events in transfusion recipients might improve clinical outcomes.

In the initial TRALI mouse model, transfusion of major histocompatibility complex (MHC) I antibodies 1 to BALB/c mice resulted in TRALI induction (22). In a subsequent study, housing the mice in a specific pathogen-free barrier facility prevented the same lung injury, unless the mice were primed by treatment with lipopolysaccharide (LPS) (23). The difference in results according to housing conditions may be explained by gut flora (24). Therefore, gut flora can be considered a pivotal factor in the priming of TRALI. However, the impact of gut flora on TRALI pathogenesis requires further study.

**Figure 2:**
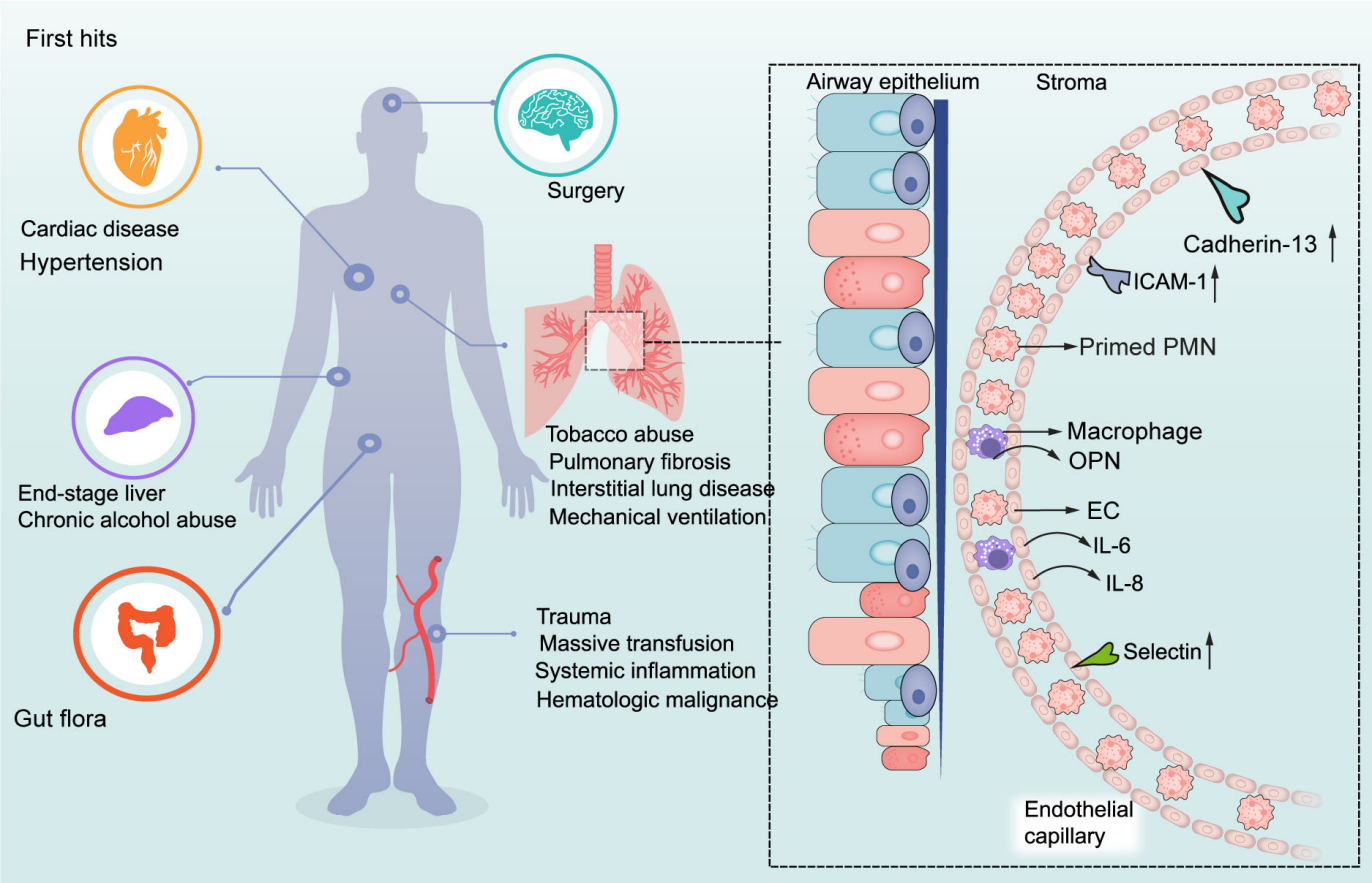
First hits in the pathogenesis of TRALI. The underlying diseases of patients stimulate the activation of ECs and macrophages, thus attracting PMNs from circulating pool to the injured lung.

The role of risk factors of recipients in TRALI pathogenesis has been investigated in preclinical studies, employing low-dose LPS or C-reactive protein (CRP) as the first hit in two-hit TRALI models to represent systemic inflammation in transfusion recipients (25, 26). However, the nature of TRALI first hits, e.g., LPS, is different from CRP. For instance, LPS activates cells by triggering a toll-like receptor four-signal pathway (27). Alternatively, CRP can represent the first hit by benefiting PMN sequestration in the lungs and activating ECs (27). Collectively, these first hits facilitate the activation of pulmonary ECs and macrophage to release interleukin (IL)-6, macrophage inflammatory protein-2 (MIP-2), the murine homolog of human IL-8, and osteopontin (OPN) and increase the expression of adhesion molecules and cadherin-13 due to endothelial activation (14, 26, 28, 29) (**Figure 2**). Circulating PMNs retained within the lung microvasculature constitute a lung-marginated pool that maintains a dynamic equilibrium with the circulating pool (30). These elevated molecules attract PMNs from the circulating pool to travel to the injured lung and firmly adhere to ECs, creating a predisposition for developing TRALI (**Figure 2**). These mechanisms might account, in part, for why patients with underlying clinical conditions, such as systemic inflammation, are at a high risk of developing TRALI. However, multiple underlying conditions that are not associated with systemic inflammation, such as chronic alcohol abuse and hypertension, among others, also represent high-risk factors for TRALI onset. In particular, reduced production of nitric oxide, an endothelial anti-adhesive molecule, strengthens endothelial intercellular cell adhesion molecule-1-dependent PMN adhesion, thus providing an alternate mechanism for PMN priming in the absence of systemic inflammation (31).

Taken together, these data indicate that endothelial activation and pulmonary sequestration are key process in the priming step, where direct activation of pulmonary ECs leading to PMN priming initiates the first phase of TRALI development.

### 2.2 Pulmonary reaction

Following pulmonary endothelial activation and PMN priming caused by underlying diseases, donor-derived risk factors, including pathogenic antibodies and BRMs, target different cell populations, such as hematopoietic and non-hematopoietic cells. Thus, these risk factors make specific contributions to the host response and uniquely influence TRALI progression (13, 14), representing the pulmonary reaction phase.

**Figure 3:**
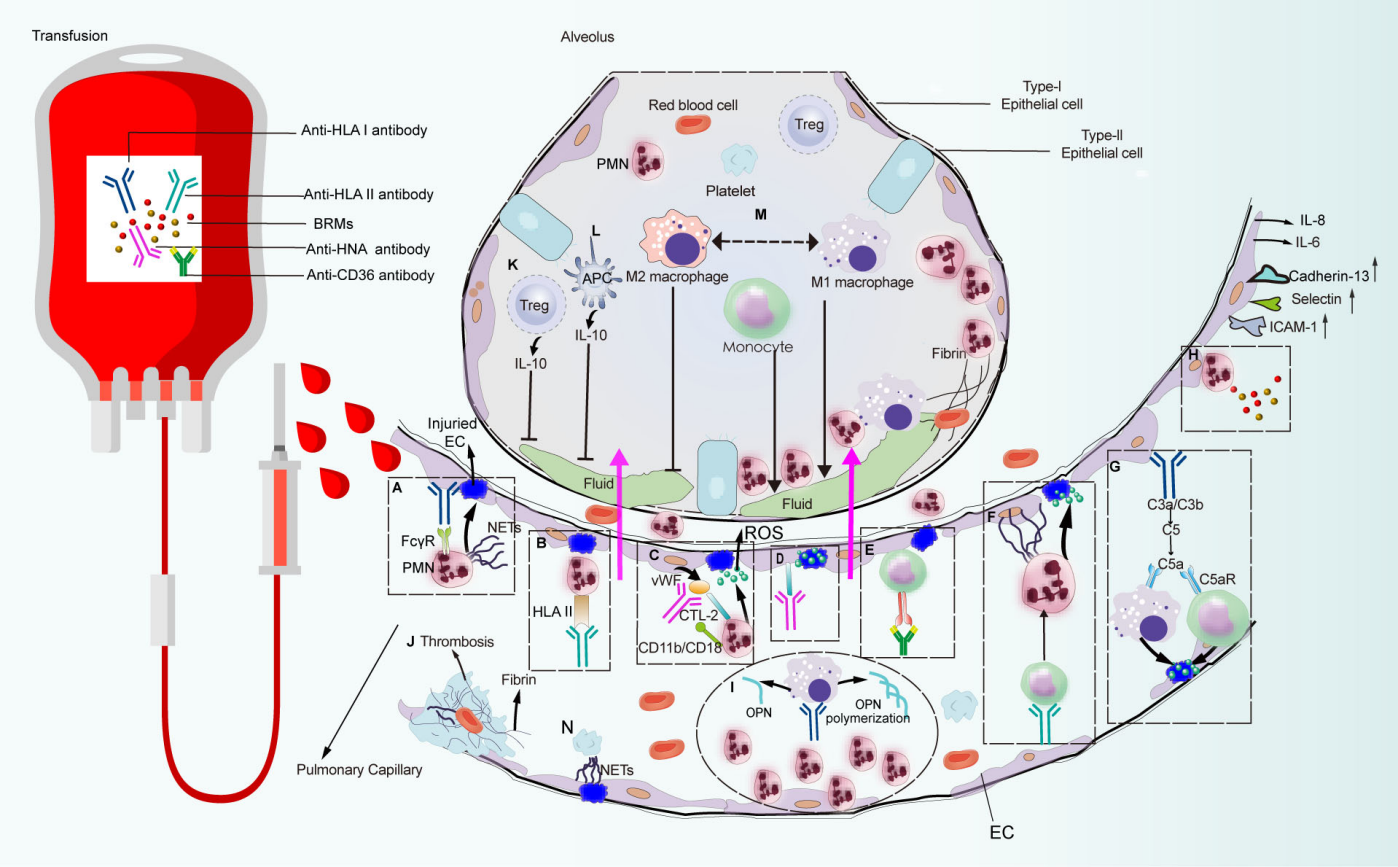
Pulmonary reaction and effector phase of TRALI pathogenesis. Pathways that are constituted with detrimental factors from blood products which connect with immune cells and non-hematopoietic cell lead to pulmonary edema or lung injury alleviation. Pathways **(A–N)** are systematically discussed in the main text. CTL-2, choline transporter-like protein-2.

In wild-type BALB/c or C57BL/6 mice, the development of pulmonary edema requires monocytes/macrophages, while PMNs and platelets appear to be dispensable. Conversely, regulatory T cells (Tregs) and dendritic cells (DCs) are associated with lung injury amelioration (32). Meanwhile, given that ECs represent the key regulator of PMN transmigration, they may represent pivotal targets for TRALI induction (33). However, the interplay between pathogenic antibodies and their target cells remains unclear in the context of antibody-dependent TRALI pathogenesis. In this section, we discuss how antibodies or BRMs in transfused blood components respond to target cells and how the induced aberrant host response delineates the different pathogenic scenarios of TRALI (**Figure 3**).

#### 2.2.1 PMNs

Based on patient autopsy reports and preclinical studies on the TRALI reaction, PMNs are regarded as the main pathogenic cells mediating lung injury (13, 26). Among the initially reported TRALI cases, anti-HLA class antibodies were the most frequently identified antibody types in blood products, of which anti-HLA class I antibodies were the most prevalent (21, 34). Indeed murine models that mimic human anti-HLA class I antibody-dependent TRALI—administration of anti-MHC class I mAbs (clone 34–1–2S) against murine H2K^d^, or the H-2^d^ antigen—has advanced our understanding of this disease. The essential role of PMNs in TRALI was first identified in preclinical models, in which *in vivo* ablation of PMNs reportedly protected mice from anti-MHC-I-mediated TRALI development (23, 32). In the first genetically manipulated *in vivo* murine TRALI model, endothelial-bound 34-1-2S reacted with the pulmonary PMN Fc gamma receptor (FcγR), leading to PMN activation and lung injury (22) (**Figure 3A**). Although resident PMNs do not regularly express HLA class II, high levels are expressed on the surface of activated PMNs (35). Moreover, evidence from two-hit rat models revealed that transfusion of donor antibodies against HLA class II stimulated PMN activation and TRALI induction (35, 36) (**Figure 3B**). In a rat anti-HNA-2a-mediated TRALI model, the antibody directly activated PMNs in the presence of cognate antigen on the surface of PMNs in an EC-independent manner (37). Unlike other anti-leukocyte antibodies that participated in TRALI development, anti-HNA-3a antibodies failed to induce direct PMN activation and appeared to primarily interact with ECs (38) (**Figure 3D**). Following the activation of ECs, von Willebrand factor (vWF) was produced and released into circulation; subsequently, anti-HNA-3a antibodies bound to the trimolecular complex comprising choline transporter-like protein-2, vWF, and CD11b/CD18 on the surface of PMNs, leading to PMN activation and agglutination *via* CD11b/CD18 signal transduction, which may promote TRALI-associated endothelial leakage (38) (**Figure 3C**).

In the case of antibody-independent TRALI, lyso-PCs generated during blood storage caused PMN-mediated EC leakage in an *in vitro* TRALI model, in which human pulmonary microvascular endothelial cells (HPMVECs) were primed with LPS and co-cultured with PMNs followed by the addition of lyso-PCs as the second hit (39) (**Figure 3H**). CD40—the CD40L receptor—is distributed on the surface of leukocytes, platelets, and ECs (40). The interaction between CD40 and CD40L has been proposed as a proinflammatory feature and a pivotal pathogenic response to inflammation and organ injury (41). Stored platelet microparticles that released sCD40L boosted PMN-dependent HPMVEC damage, which may contribute to TRALI onset (40). Indeed, blocking the CD40-CD40L interaction with an anti-CD40L antibody protected mice against 34-1-2S-mediated TRALI development by suppressing PMN migration into the alveolar space (42, 43).

**Figure 4:**
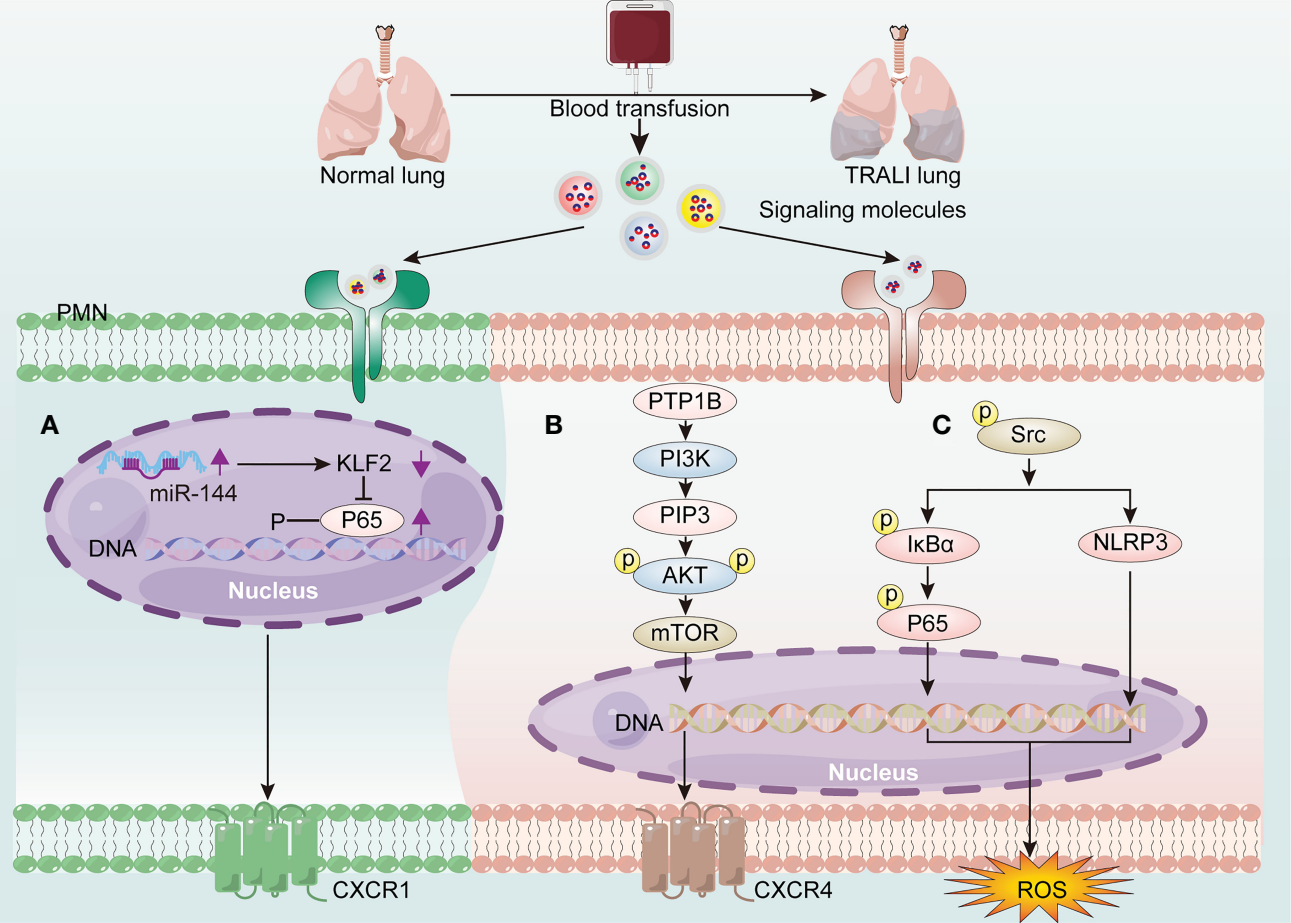
The molecular mechanisms of driving PMN activation in TRALI pathogenesis. Blood transfusion induces PMN activation *via* the generation of signaling molecules activating the receptors on PMNs, thereby exacerbating TRALI. **(A)** Up-regulated miR-144 promotes PMN activation through activating the NF-κB/CXCR1 signaling pathway *via* down-regulation of KLF2. **(B)** PTP1B activates PMNs *via* PI3Kγ/AKT/mTOR-dependent CXCR4 signaling. **(C)** HLA-A2 induces PMN-derived ROS production *via* activating NF-κB/NLRP3 inflammasome *via* phoosphorylate-Src elevation.

Although the aberrant activation of PMNs is recognized as the main feature of TRALI, the underlying molecular mechanisms driving PMN activation remain elusive. MicroRNAs (miRNAs)—single-stranded non-coding RNAs constituting approximately 22 nucleotides—contributed to lung injury by interacting with the 3′-untranslated region of mRNA (44). More specifically, miR-144 caused 34-1-2S-mediated TRALI by activating the NF-κB/CXCR1 signaling pathway *via* KLF2 in a PMN-dependent manner (45) (**Figure 4A**). Additionally, protein tyrosine phosphatase-1B (PTP1B) activated the PI3Kγ/AKT/mTOR-dependent CXCR axis, which may be involved in PMN activation (46) (**Figure 4B**). Anti-human leukocyte antigen-A2 (HLA-A2) antibody can function as an initiator of TRALI (47). *In vitro* coculture of anti-HLA-A2 antibody with PMNs resulted in PMN activation and subsequent endothelial permeability (48). The anti-HLA-A2 antibody may activate PMNs by stimulating NF-κB/NLRP3 inflammasome activation *via* increased abundance of phosphorylated-Src, thus aggravating TRALI (49) (**Figure 4C**).

#### 2.2.2 ECs

Anti-leukocyte antibodies are recognized as the main contributor to TRALI pathogenesis; however, the critical binding site of these antibodies remains unclear. HLA expression on nonleukocytic cells suggests ECs as probable targets for antibody binding. As early as 2011, infusion of 34-1-2S to a murine model of human TRALI suggested that the reaction between the antibody and MHC class I molecules on pulmonary ECs represented a probable initiating event for TRALI (50). This may be explained by the high expression of MHC-I on the surfaces of murine pulmonary ECs (51). Similarly, high pulmonary endothelial MHC-I expression has been observed in clinical TRALI (51). Given that the lung capillaries represent the first location encountered by the infused antibodies, the ECs may serve as a key site for antibody binding. Furthermore, targeted deletion of endothelial MHC-I alleviated 34-1-2S-mediated lung injury, while its restoration rendered mice susceptible to lung edema (51). Additionally, the interaction between 34-1-2S and pulmonary ECs may drive the production of complement component 5a (C5a) *via* activation of the complement cascade, thus contributing to TRALI development (50). Collectively, these findings highlight the importance of 34-1-2S engagement with endothelial MHC-I in the induction of TRALI.

ECs also express other leukocyte antigens, including HLA class II and HNA-3, the interaction of which, with their respective antibodies, reportedly contributed to TRALI development, i.e., alloantibodies against HNA-3a, not HNA-3b, have been linked to severe TRALI (52, 53). Anti-HNA-3a antibodies interacted with cognate antigens on the surface of pulmonary ECs in a PMN-independent manner, thereby precipitating TRALI (54, 55).

Moreover, ECs participate in antibody-independent TRALI *via* direct endothelial dysfunction. Transfusion of aged platelets elicited TRALI *via* acid sphingomyelinase (ASM)-forming ceramide-mediated endothelial apoptosis, further accelerating endothelial barrier failure (56). Furthermore, stored platelet-derived injurious ceramide enhanced the formation of extracellular vesicles in an ASM-dependent manner, which transported ceramide to the lung, resulting in endothelial barrier failure and TRALI onset (57, 58). Moreover, red blood cell (RBC) transfusion was a hazard factor for the induction of pulmonary respiratory failure (59, 60). RBC transfusion-mediated lung injury may be related to pulmonary ECs undergoing necrotic cell death, i.e., necroptosis (61, 62).

Collectively, these data support the evidence that ECs may be crucial regulators of TRALI.

#### 2.2.3 Monocytes/macrophages

The binding of anti-leukocyte antibodies to corresponding cognate antigens distributed on PMNs or pulmonary ECs may lead to PMN activation and induction of lung injury. However, given that PMN depletion did not prevent TRALI development in murine antibody-mediated TRALI (54), other cells have also been implicated. Although HLA class II antigen is not specifically expressed on PMNs or ECs, anti-HLA class II antibodies are consistently involved in TRALI onset. Indeed, plasma containing anti-HLA class II antibodies effectively sensitized peripheral blood mononuclear cells, leading to severe transfusion reactions *via* the production of inflammatory cytokines, such as IL-1β and IL-6, and chemokines (IL-8) by monocytes (63). Moreover, evidence from an *ex vivo* model showed that anti-HLA class II antibody-initiated monocyte activation was attributable to exaggerated HPMVEC permeability, which reflected endothelial damage in patients suffering from TRALI (64). Consistent with this result, anti-HLA class II antibody-containing plasma can cause matched monocyte activation, resulting in the initiation of a PMN amplification cascade and lung endothelial damage in an *in vitro* two-hit model (65) (**Figure 3F**). This finding has been further verified in an *ex vivo* lung model, which showed that anti-HLA class II antibody-mediated TRALI was dependent on monocytes, while PMNs were apparently dispensable (65). Following injection of 34-1-2S to severe combined immunodeficient (SCID) mice, the intact antibody reacted with its cognate antigen on peripheral blood monocytes, resulting in monocyte-dependent upregulation of MIP-2 expression, leading to recruitment of PMNs to the lung microvasculature, where the Fc domain of the antibody led to full TRALI initiation (66). Additionally, in place of FcγR-mediated PMNs activation, the C5a receptor on the surface of peripheral blood monocytes/macrophages responded to C5a derived from complement cascade activation, causing disease induction (50) (**Figure 3G**). Furthermore, individuals with CD36-deficient platelets and monocytes can develop isoantibodies against CD36 because of incompatible immune stimulation, which contributes to TRALI onset (67–69). The preliminary mechanism may be associated with anti-CD36 antibody-Fc receptor (FcR) binding to monocytes, thus inducing the TRALI reaction (70) (**Figure 3E**).

Macrophages have also been identified as major opsonins involved in TRALI induction. *In vivo* macrophage-derived OPN polymerization was involved in PMN chemotaxis (71), whereas macrophage elimination protected mice from antibody-mediated TRALI (26) (**Figure 3I**). Macrophages exert diverse functions, facilitated in part by their ability to undergo polarization (72), which enables them to readily adopt phenotypes based on stimulation within their microenvironment (73). Macrophages are roughly categorized as M1 or M2 according to phenotype. M1 macrophages are regarded as the proinflammatory phenotype with microbicidal potential, while M2 macrophages have a propensity to facilitate tissue repair and cell proliferation (72). Using a murine TRALI model, Wang et al. (74) confirmed that M1-polarized alveolar macrophages (AMs)—the proinflammatory phenotype—played a critical role in lung injury. Lung edema in TRALI mice can be abrogated by inhibiting the polarization of AMs toward the M1 phenotype (74) (**Figure 3M**).

These data suggest a clear role for monocytes/macrophages in TRALI pathogenesis.

#### 2.2.4 Recipient platelets

Platelets derived from bone marrow megakaryocytes are commonly associated with thrombosis and hemostasis (75). However, beyond these traditional roles, platelets are key effectors of thromboinflammation and immune responses *via* crosstalk with various immune cells, such as PMNs (76). Moreover, considering the observed thrombocytopenia in TRALI patients, as well as the results of murine antibody-mediated TRALI models, platelets appear to be drivers of TRALI (13, 77). However, the evidence regarding the role of recipient platelets in aggravating lung injury is conflicting, i.e., the role of recipient platelets as either pathogenic or dispensable, in the context of TRALI development, within murine models injected with 34-1-2S is dependent on the experimental designs for assessing lung injury. On the one hand, treatment with rabbit polyclonal anti-mouse platelet serum to eliminate platelets, as well as 100 mg kg^−1^ aspirin or tirofiban to inhibit platelet reactivity, effectively prevented alveolar edema (23, 78, 79). Moreover, GPIb—a major platelet receptor—has been implicated in platelet activation and aggregation (76). However, platelet depletion, using an anti-GPIbα antibody, improved TRALI outcomes, without influencing its progression (80). On the other hand, several studies using different animal models have shown that platelet depletion or inhibition was dispensable in TRALI development. For example, neuraminidase-induced thrombocytopenia failed to effectively protect mice from TRALI (50, 81), diphtheria toxin-induced immunologic thrombocytopenia did not prevent PF4-cre/iDTR mice from 34-1-2S-associated lung edema (82), and pretreatment of mice with ML354—a protease receptor 4 pathway inhibitor—failed to prevent TRALI induction following activation of platelets *via ex vivo* stimulation (80).

Recently, novel insights regarding recipient platelets have been reported. Receptors bound to immunoglobulin G (IgG) antibody are uniquely expressed on human platelets and include IgG receptor I (FcγRI) and FcγRIIA, among others (83). Among these receptors, FcγRIIA/CD32A is the most extensively distributed FcγR on the surface of human platelets (83). Yet, its effects on TRALI have not been reported in mouse models, suggesting that the previous conclusions regarding the role of recipient platelets as main contributors to the pathogenicity of antibody-dependent TRALI are potentially incomplete. To address this gap in knowledge, EI Mdawar et al. (84) established an FcγRIIA/CD32A transgenic mouse model. When the humanized mouse model expressing the FcγRIIA/CD32A receptor was challenged with 34-1-2S, aggravated lung edema was observed (84). The authors concluded that the associated pathogenesis was caused by TRALI induction triggering platelet activation and subsequent 5-hydroxytryptamine 2A serotonin release. Thus, recipient platelets appear to represent key effector cells in exaggerated lung injury; however, their contribution to TRALI onset and pathogenesis requires further investigation.

#### 2.2.5 DCs/Tregs/IL-10

Conflicting evidence has been reported regarding the role of recipient T cells in other inflammatory lung injury models. For example, T-cell activation may aggravate endotoxin-induced acute lung injury (ALI) (85). Meanwhile, mice treated with LPS developed ARDS, whereas lethal lung injury was significantly ameliorated by decreasing CD3+ T cells *in vivo* (86). Conversely, murine ARDS induced by reovirus was critically dependent on T cells and their ability to secret interferon-γ (17). In contrast, reconstituted T cells protected SCID mice from lung injury, implicating T cells as protective factors against TRALI development (17). This was further confirmed *via* removal of Tregs, DCs, and IL-10, resulting in murine TRALI initiation (32), which was likely related to the protective effect elicited by Treg- and DC-secreted IL-10. Indeed, both patients with low levels of IL-10 and mice lacking IL-10 were susceptible to TRALI development (32, 87). Hence, the Treg-DC-IL-10 axis has been proposed as a protective mechanism against TRALI induction (**Figures 3K, L**). However, to date, the mechanism underlying the protective effect of IL-10 remains unclear and warrants further investigation.

### 2.3 Effector phase

In this phase, activated effector cells, including PMNs, ECs, monocytes/macrophages, and platelets, produced effector molecules, such as NETs (**Figures 3A, N**) and ROS (**Figures 3C**, **D**, **F**, **G**), which directly damage pulmonary vascular ECs, resulting in lung edema. Additionally, EC dysfunction may aggravate pulmonary vascular leakage. Moreover, dysregulated coagulation has been implicated in lung injury, including that associated with TRALI and severe acute respiratory syndrome coronavirus 2 (SARS-CoV-2) mediated ALI (77, 88).

#### 2.3.1 NETs

NETs can effectively restrict pathogen spreading and kill microorganisms *via* the release of high doses of histones and granule contents by active immune cells (89). The role of NETs in TRALI was first reported in 2012 by Thomas, who observed NETs in the blood of patients and mice with TRALI (81). They further observed that PMN-derived NETs were released *in vitro* following challenge by an anti-leukocyte antibody. More recently, the formation of NETs *in vivo*, as well as their detrimental contributions, have been further validated in TRALI models (90, 91). The complement system has also been shown to function as a key trigger of NET production by assisting the 34-1-2S in PMN activation (51, 92). However, NETs are not exclusively produced by PMNs. Platelet-derived NETs can directly damage pulmonary ECs, further aggravating TRALI (93). Moreover, within a murine TRALI model, an increase in platelet–neutrophil interactions and NET formation was observed, with fewer NET produced following the administration of platelet-targeting therapeutics (78). Additionally, NET formation has been observed in antibody-independent TRALI. For example, under long-term storage, RBC hemolysis can occur and cause sufficient hemin accumulation in the solution (94). Hemin induced NET release, resulting in TRALI induction (94). Hence, NETs function as pivotal effector molecules of TRALI induction.

#### 2.3.2 ROS

Upon exposure to inflammatory factors, the NADPH oxidase family members are activated, resulting in the production and release of a large amount of ROS, leading to lung disease (95). ROS are important effector molecules involved in TRALI pathogenesis. That is, trapped PMNs within the lung capillary bed being activated by anti-leukocyte antibodies and BRMs from transfused blood components, resulting in the production and release of ROS, which further destroy the ECs, causing capillary leakage and pulmonary edema (48, 96). PMNs are also recruited to the lungs, where they produce ROS that damage the ECs (26). Thus, PMN-derived ROS production blockade was an effective strategy for protecting mice from antibody-mediated TRALI (50, 97, 98). Activated monocytes/macrophages and pulmonary ECs also generated ROS, thus inducing deterioration of endothelial permeability and TRALI onset (50, 54). More importantly, gp91phox-KO mice, which lacked the ability to induce ROS, were protected from antibody-mediated TRALI, indicating that ROS were essential for TRALI induction (32). Storage of blood products can cause vesicle shedding and accumulation of hemin and lysoPC, which contributed to aberrant oxidative bursts, ROS production, and lung endothelial damage *in vitro* (39, 99–101). This may represent a potential pathogenesis for antibody-independent TRALI development.

#### 2.3.3 Imbalanced coagulation and the fibrinolysis system

Microthrombosis occured in the pulmonary vascular walls of animals with TRALI (84). Hence, considering that ECs are the key initiator and regulator of coagulation (102), coagulopathy may contribute to deteriorated lung function following transfusion of pathogenic antibodies or BRMs that would destroy pulmonary ECs. Indeed, coagulopathy has been identified as a pivotal pathogenic characteristic in rat models of aged erythrocyte- or platelet-mediated TRALI (103, 104). A case-control study reported that blood transfusions in patients who underwent cardiovascular surgery and exhibited obvious pulmonary inflammatory reactions were accompanied by coagulation and fibrinolysis dysregulation, leading to worsened prognosis in the ICU (105), thus further demonstrating the potential role of coagulopathy in TRALI development. Moreover, intravital lung imaging revealed platelet adhesion to the pulmonary ECs as well as occasional thrombotic events in a murine 34-1-2S-mediated TRALI model (51). Coagulopathy was involved in the dysregulation of coagulation and fibrinolytic pathways (106), leading to fibrin deposition in the lungs (77). Fibrin effusion in the lung alveoli was recognized as a main contributing factor to ALI and ARDS pathogenesis (107–109). Additionally, in a murine 34-1-2S-mediated TRALI model, increased coagulation, impaired fibrinolysis, and fibrin deposition were observed in the lungs (77) (**Figure 3J**). In fact, fibrin deposition in the lungs may activate ECs, thereby promoting the release of proinflammatory mediators and increasing vascular permeability and lung injury (77).

## 3 Management

The morbidity associated with TRALI has plummeted with the introduction of mitigation strategies that include donor management, blood component processing, and patient blood management (PBM). However, no specific therapies are available and therapeutic options primarily focus on supportive measures. Hence, additional research is needed to develop, standardize, and evaluate novel drugs for the treatment of TRALI. Recently, a full spectrum of potential targets, including anti-inflammatory and immunoregulatory factors, have been investigated as potential TRALI therapeutics. Recent progress in prevention strategies (**Table 1**) and treatments (**Table 2**) has been made and is described below.

**Table 1:** Summary of the prevention strategies of TRALI.

Disease	Strategy			Type of study	Effects	Country	Year	Sources
Antibody dependent TRALI	Donor management		Male-only plasma	Retrospective study	Decline in TRALI	Australia	2022	Sivakaanthan et al. (21)
				Retrospective study	No TRALI cases reported	UK	2017	Bolton-Maggs et al. (110)
			Male-dominate plasma	Retrospective study	Decline in TRALI	Australia	2022	Sivakaanthan et al. (21)
			Exclusion of allo-exposure donors	Prospective study	Decline in TRALI	Netherlands	2012	Middelburg et al. (111)
			Screening for antibodies	Retrospective study	Decline in TRALI	Germany France UK Sweden USA	2012	Reesink et al. (112)
	SDP			Clinical study	No cases of TRALI	Norway	2003	Flesland et al. (113)
				Systematic review and meta-analysis	No cases of TRALI	Netherlands	2017	Saadah et al. (114)
				Clinical study	No cases of TRALI	Netherlands	2020	Saadah et al. (115)
				Retrospective study	TRALI occurrence	Netherlands	2022	Klanderman et al. (20)
				Case series	TRALI occurrence	Netherlands	2022	Klanderman et al. (116)
Antibody independent TRALI	Leukoreduction			Retrospective study	Decline in TRALI	USA	2010	Blumberg et al. (117)
				Preclinical study	TRALI mitigation	USA	2014	Silliman et al. (47)
				Preclinical study	TRALI mitigation	USA	2020	McQuinn et al. (118)
				Systematic review	Invalid in prevention TRALI	Ecuador	2015	Simancas-Racines et al. (119)
	PRT	Mirasol		Preclinical study	Invalid in prevention TRALI	USA	2010	Silliman et al. (120)
		Mirasol		Preclinical study	Incapable of inducing TRALI	USA	2015	Caudrillie et al. (121)
		Mirasol		Preclinical study	Incapable of inducing TRALI	USA	2017	Mallavia et al. (122)
		Mirasol		Retrospective study	Valid in prevention TRAL	Ghana	2019	Owusu-Ofori et al. (123)
		Intercept ^(TM)^		Prospective study	No TRALI cases	Italy	2015	Knutson et al. (124)
		Intercept ^(TM)^		Cohort study	No effect on prevention TRALI	USA	2022	Snyder et al. (125)
	PAS			Review	Reducing the incidence of TRALI	USA	2019	Kuldanek et al. (126)
				Review	Reducing the incidence of TRALI	Netherlands	2018	van der Meer et al. (127)
TRALI	PBM			Review	Decreasing the incidence of TRALI	USA	2017	Friedman et al. (128)
				Retrospective study	Decline the incidence of TRALI	Austria	2019	Tung et al. (129)
				Retrospective study	Avoiding TRALI occurrence	Sri Lanka	2022	Priyanjani et al. (130)

TRALI, transfusion-related acute lung injury; SDP, solvent/detergent treated pooled plasma; PRT, pathogen reduction technology; PAS, platelet additive solution; PBM, patient blood management.

**Table 2:** Summary of innovative treatments undergoing TRALI.

Disease	Targeting	Strategy	Delivery Route	Treatment Regiment	Results/ Outcomes	Proposed mechanism of action	Type of study	References
TRALI	Supportive care	Oxygen inhalation	Not mentioned	Not mentioned	Relieving respiratory distress	Maintaining hemodynamics	Review	Semple et al. (13), 2019
							Review	Kuldanek et al. (126), 2019
		Ventilation	Invasive	Not mentioned			Review	Kuldanek et al. (126), 2019
			Noninvasive	Not mentioned				
		Extra-corporeal membrane oxygenation	Intravenous catheterization	Not mentioned			Review	Kuldanek et al. (126), 2019
				Lasting 15 h after the operation			Case reports	Honda et al. (131), 2015
	Serum proinflammatory, anti-inflammatory markers levles, and oxidative stress	Ascorbic acid	I.V.	2.5 gm/6 h, 96 h	Better 7-days survival	Reduced most proinflammatory markers levels and oxidative stress; elevated anti-inflammatory marker	Clinical trial	Kassem et al. (132), 2022
	Gut glora	Broad spectrum;(vancomycin, ampicillin, neomycin, and metronidazole)	Oral	1 mg/mL, every 48 hours for 1 week	Preventing murine TRALI	Decreasing plasma MIP-2 levels and pulmonary PMN recruitment	Preclinical study	Kapur R et al. (24), 2018
Antibody-dependent TRALI	NETs	Aspirin	I.P.	100 μg/g, 30 min prior to LPS priming and again 2 h prior to MHC-I mAb challenge.	Alleviating lung injury	Decreasing NET formation and NET-associated platelets sequestration	Preclinical study	Caudrillier et al. (78), 2012
		DNase1	I.V.	10 mg/kg, at the same time of H2Kd mAb injection; 5 minutes after H2Kd mAb injection	Alleviating lung injury	Decreasing NET formation and NET-associated platelets sequestration	Preclinical study	Caudrillier et al. (78), 2012
		DNase1	Intranasal	50 μg/mouse, 10 min before or 90 min after H2Kd antibody injection.	Improving arterial oxygen saturation	Disrupting NET accumulation	Preclinical study	Thomas et al. (81), 2012
		Disulfiram	I.P.	50 mg/kg, 24 h and 3 h before H2Kd antibody injection.	Improving survival	Blockade of NET formation	Preclinical study	Adrover et al. (91), 2022
		MSI-1436	I.P.	10 mg/kg, 2 h before TRALI induction	Preventing murine TRALI and improving survival	Limiting NET production	Preclinical study	Song et al. (46), 2022
	ROS	IVIg	I.P.	2 g/kg, 18 h before 34-1-2S injection; 1 g/kg dose of IVIg 3 min post 34-1-2S injection	Preventing TRALI as well as reducing lung injury	Inhibiting PMN derived ROS production	Preclinical study	Semple et al. (97), 2012
	PMNs	MSI-1436	I.P.	Optimal dose of 10 mg/kg, 2 h before 34-1-2S injection.	Preventing murine TRALI and improving survival	Promoting PMN aging	Preclinical study	Song et al. (46), 2022
		Dasatinib	Oral	Not mentioned	Alleviating lung injury	Inhibited PMN activation	Preclinical study	Le et al. (49), 2022
	ECs	IL-35	I.V.	100 μg/kg, once a day for 2 days before the TRALI model, and the model was generated on the third day, with a third injection before 34-1-2S injection.	Preventing TRALI	Inhibition of endothelial activation	Preclinical study	Qiao et al. (133), 2020
	Macrophage	anti-OPN antibody	I.V.	2.25 mg/kg, injected with TRALI induction antibodies	Preventing TRALI onset	Blocking OPN derived from macrophages	Preclinical study	Kapur et al. (26), 2019
		AAT	Exogenous gene delivery; hydrodynamic injection	A mixture of a pattB-CMV-AAT and a mouse codon-optimized PhiC31o vector was codelivered to hepatocytes by hydrodynamic injection	Alleviating lung injury	Suppressing AMs polarization toward the proinflammatory M1 phenotype	Preclinical study	Wang et al. (74), 2020
	Platelets	Sarpogrelate	I.V.	1 mg/kg,5min before 34-1-2S injection; injection after TRALI induction	Abolishing lung edema	Blockade of platelet FcγRIIA/CD32A activation mediated serotonin secretion	Preclinical study	El Mdawar et al. (84), 2021
		Tirofiban	I.V.	2 μg/g, 30min before 34-1-2S injection	Decreasing lung injury	Suppressing platelet activation and targeting pulmonary coagulation-fibrinolytic disorders	Preclinical study	Yuan et al. (134), 2023
	Anti-inflammatory cytokine	IL-10	I.V.	45 mg/kg, together with the TRALI induction antibodies or 15 min after injection of TRALI induction antibodies	Protecting and rescuing murine TRALI	Removal of a major anti-inflammatory brake.	Preclinical study	Kapur et al. (32), 2017
	Treg	IL-2/IL-2c	I.P.	IL-2 5μg/kg or IL-2c (1 mg of recombinant murine IL-2 and 10 mg of mouse IL-2 antibody) administered to mice for 5 consecutive days before TRALI model induction	Preventing murine TRALI	Activation of Treg-IL-10 axis	Preclinical study	He et al. (135), 2019

TRALI, transfusion-related acute lung injury; h, hour; I.V., intravenous injection; I.P., intraperitoneal injection; min, minute; MIP-2, macrophage inflammatory protein-2; NETs, neutrophil extracellular traps; ROS, reactive oxygen species; mAb, monoclonal antibody; IVIg, intravenous immunoglobulin; PMNs, polymorphonuclear neutrophils; ECs, endothelial cells; IL, interleukin; OPN, osteopontin; α1-antitrypsin, AAT; AM, alveolar macrophage; FcγR, IgG receptor; IL-2c, anti-IL-2 complexes; Treg, regulatory T cell.

### 3.1 Prevention

#### 3.1.1 Antibody-mediated TRALI prevention strategies

##### 3.1.1.1 Donor management

Considering that anti-HLA or anti-HNA antibodies are detected most frequently in multiparous donors, TRALI risk-reduction strategies, including the introduction of male-only donors, male-dominated plasma, exclusion of all-exposure donors, and antibody screening, have been introduced, resulting in a significant reduction in the morbidity associated with antibody-dependent TRALI in developed countries (4, 21, 110–112). However, anti-leukocyte antibody screening has not yet been routinely implemented because of financial complexities (136). Despite the use of these prevention strategies, additional research is needed, as anti-HLA antibodies were reportedly triggered in coronavirus disease 19 convalescent plasma donors and SARS-CoV-2 vaccination volunteers (137, 138). Therefore, TRALI prevention may also require screening antibodies against HLA antigens in donors following SARS-CoV-2 sensitization.

##### 3.1.1.2 Solvent/detergent-treated pooled plasma

Solvent/detergent-treated pooled plasma (SDP) is manufactured by treating large pools of plasma with solvent detergent to ensure that the anti-leukocyte antibody levels are below the limit of detection (116). Since the adoption of SDP in Norway in 1993, no TRALI cases were reported over a 10-year period (113). As of 2020, no TRALI cases had been reported in association with SDP transfusion (115). However, in 2022, a definite TRALI case was observed following SDP transfusion (20, 116), resulting in a total of three reported TRALI cases following SDP transfusion to date (116). Although implementation of SDP transfusion has markedly reduced the incidence of TRALI, medical staff associated with SDP transfusions must realize the potential risk regarding severe complications, i.e., TRALI.

#### 3.1.2 Antibody-independent TRALI prevention strategies

##### 3.1.2.1 Leukoreduction

Leukoreduction is a procedure that intentionally filters leukocytes in donated samples to decrease the abundance of mediators derived from leukocytes and platelets, thereby diminishing transfusion adverse reactions (117, 119). A retrospective analysis revealed that leukoreduction of blood components reduced the occurrence of TRALI cases by 83% (117). Furthermore, previous *in vivo* animal studies demonstrated that TRALI could be attenuated by leukoreduction before storage because of the reduction of proinflammatory mediators (47, 118). Conversely, leukoreduction of packed RBC failed to efficiently prevent TRALI, as reported in a systematic review (119). Hence, the effect of blood component leukoreduction on TRALI incidence should continue to be monitored.

##### 3.1.2.2 Pathogen reduction technology

The pathogen reduction technology (PRT) of Mirasol and Intercept^(TM)^ has been proven effective in improving blood component safety by targeting pathogen nucleic acid or membrane lipid structures in donor samples (124, 139). However, the efficiency of PRT to reduce the risk of TRALI has been proven inconsistent in preclinical studies. An earlier *in vivo* study reported that blood products treated with Mirasol PRT failed to prevent rat TRALI (120). However, transfusion of Mirasol PRT-treated platelets has also prevented lung injury in immunodeficient mice (121). Meanwhile, transfusion of aged Mirasol PRT-treated RBC did not deteriorate this disease in a murine TRALI model parallel with standard-delivery RBC (122). These contradictory *in vivo* results may be because of discrepancies in experimental design.

The role of PRT in TRALI risk-reduction was also debated in clinical studies. An open label, prospective hemovigilance program confirmed that no TRALI cases occurred following transfusion with Intercept^(TM)^-processed platelets (124). Similarly, data from a hemovigilance system involving 2181 transfusion records in Ghana demonstrated that Mirasol PRT-treated whole blood effectively prevented TRALI (123). However, a recent open label, sequential cohort study found that ARDS-associated morbidity did not differ significantly between patients receiving Intercept ^(TM)^ PRT platelets and those receiving conventional platelets (125). Therefore, comprehensive clinical trials are required to validate the effect of PRT blood components on TRALI risk-reduction.

##### 3.1.2.3 Platelet additive solution

Following the introduction of risk-reduction strategies in 2014, the rate of TRALI did not differ between blood components (140). However, a significantly higher incidence of TRALI was sustained in female-donated platelets compared with that in male-donated platelets (140), which may be because of the platelets suspended in female plasma containing anti-HLA or anti-HNA antibodies. Meanwhile, buffy-coat-derived platelets suspended in platelet additive solution (PAS) can reduce TRALI-associated morbidity, which was similar to that reported in single-donor platelets (126, 127). Thus, additional strategies that may decrease the incidence of TRALI must be implemented, including replacement of traditional residual plasma with novel PAS in buffy coat pooled platelets.

#### 3.1.3 TRALI PBM

PBM attempts to optimize the patient’s hematologic capabilities, decrease bleeding, and limit unnecessary transfusions (141). Findings from the hemovigilance network in the United States in 2015 highlighted the ability of PBM to decrease the incidence of TRALI (128). Similarly, a decrease in TRALI incidence was observed in Austria following the introduction of PBM (129). A recent study further emphasized that proper PBM represented the standard tool for avoiding TRALI occurrence (130).

### 3.2 Treatment

#### 3.2.1 Supportive care

To date, there are no established treatments for TRALI beyond supportive care, which comprises oxygen inhalation, ventilation, and extra-corporeal membrane oxygenation to effectively maintain hemodynamics (13, 126, 131). In rare cases of sickle cell disease, patients experiencing TRALI also receive 5% albumin, erythropoietin, and iron as supportive modalities (142). Although pulmonary edema is common, diuretics should be avoided because of the associated hypotension (143).

#### 3.2.2 Clinical studies

Ascorbic acid, a water-soluble vitamin, can limit ROS damage to the epithelial barrier. A randomized controlled trial revealed that critically ill patients with TRALI exhibited a better 7-day survival following intravenous administration of high-dose ascorbic acid (132). To fully explore more effective TRALI treatments, future large-scale multi-center randomized controlled trials and in-depth clinical studies are needed.

#### 3.2.3 Preclinical studies

Activation of ECs, PMNs, monocytes/macrophages and platelets leading to effector molecule production and pulmonary fibrin deposition is the key process of the onset of pulmonary edema, the hallmark of TRALI. Hence, blocking these pathways may effectively prevent TRALI initiation. Additionally, targeting gut flora and Tregs/IL-10 seems to be prospective prevention strategies or treatments.

##### 3.2.3.1 Targeting gut flora

Since gut flora resulted in mice susceptibility to 34-1-2S-dependent TRALI, depletion of gut flora in mice may be a promising strategy for relieving TRALI. Utilizing broad-spectrum antibiotics composed of vancomycin, ampicillin, neomycin, and metronidazole in drinking water to kill gut flora can prevent TRALI in mice *via* decreasing plasma MIP-2 levels and PMN recruitment in the lungs (24).

##### 3.2.3.2 Targeting effector molecules

Various compounds have been found to halt the effect of NETs. For instance, DNase I is an effective therapeutic drug for digesting NET present in the lungs (91). Combined aspirin and DNase1 treatment reduced NET formation and NET-associated platelet sequestration, thus alleviating TRALI (78). Moreover, intranasal DNase 1 treatment of mice before 34-1-2S infusion, or injection 90 min after TRALI occurrence, can disrupt NET accumulation in the alveoli and improve blood oxygenation (81). Meanwhile, inhibition of NET formation is also an effective means to prevent TRALI. Disulfiram—an existing FDA-approved drug for alcohol use disorder—effectively improved the survival of murine 34-1-2S-induced TRALI *via* blockade of NET formation (91).

Intravenous immunoglobulin (IVIg) is biologic components of polyclonal antibodies, extracted from human plasma of large healthy donor cohort blood banks. IVIg has proven beneficial for the clinical treatment of autoimmune diseases, chronic lymphocytic leukemia, and sepsis (144). To date, IVIg therapy remains off-label for patients with TRALI. Nevertheless, one study has assessed the efficacy of IVIg in a TRALI model and found that it prevented murine antibody-mediated TRALI and reduced lung injury at the level of PMN-derived ROS production, thus improving lung injury (97). Conversely, several clinical lines of evidence have reported that IVIg may contribute to TRALI induction (145, 146). Although IVIg may contribute to TRALI induction, this is extremely rare given the amount of IVIg infused annually. Due to these controversial findings, the therapeutic pathway associated with IVIg requires further elucidation.

##### 3.2.3.3 Targeting PMNs

The pivotal role of PMNs in TRALI provides a basic principle for exploiting PMN-targeted therapies. PMN aging is described as a “programmed disarming” that decreases the capacity of PMNs to inflict damage after infiltrating target tissues (147). Based on this theory, it is proposed that therapeutic intervention to regulate PMN aging may protect against tissue damage. PTP1B regulates signaling events that are of fundamental importance to homeostatic control (46). PTP1B inhibitors, including MSI-1436 and DPM-1003, have been tested in clinical trials for obesity and metastatic breast cancer (46). Inhibition of PTP1B with MSI-1436 effectively promoted PMN aging, thus preventing murine TRALI and improving survival (46). Dasatinib—a tyrosine kinase inhibitor commonly used to improve leukemia survival rates (148)—provided a clinical benefit in PMN-mediated inflammatory diseases by influencing the proinflammatory functions of mature PMNs (148). Moreover, dasatinib inhibited PMN activation in a TRALI mouse model by negatively regulating the NF-κB/NLRP3 inflammasome activation (49).

##### 3.2.3.4 Targeting endothelial activation

As endothelial activation is crucial to TRALI onset, it may represent an effective therapeutic target for preventing lung injury caused by blood transfusion. IL-35, which belongs to the IL-12 cytokine family, is regarded as a novel immune-suppressive cytokine (149). IL-35 may alleviate disease states by inhibiting pulmonary endothelial proliferation, apoptosis, and activation (133, 150). IL-35 treatment has been proven to prevent murine TRALI *via* inhibition of endothelial activation (133), which could provide new insights into therapeutic targets.

##### 3.2.3.5 Targeting macrophages

The treatments for targeting macrophages, including blocking the production of key effectors by macrophages and regulating macrophage polarization, represent potential TRALI therapies. OPN derived from macrophages is a matricellular protein that served a crucial role in PMN migration (26). Meanwhile, inhibition of OPN with an anti-OPN antibody in the presence of macrophages can prevent TRALI onset (26). Likewise, inducing polarization of macrophages toward the anti-inflammatory M2 phenotype has improved various disease states in preclinical models (151, 152). In particular, α1-antitrypsin (AAT) elicits tissue-protective effects as well as anti-inflammatory and immunomodulatory properties (74). Application of human AAT exogenous gene delivery technology to 34-1-2S-treated mouse livers successfully alleviated lung injury *via* suppressing M1 polarization (74). Hence, targeting macrophage-associated inflammatory proteins or promoting polarization toward the M2 phenotype may serve as prospective therapeutic strategies.

##### 3.2.3.6 Targeting platelet activation

Sarpogrelate has been widely used in treating cardiovascular disorders *via* specifically antagonizing 5-HT2A receptor signaling (153). Thus, to investigate the role of 5-HT2A receptor in serotonin-mediated deterioration that caused pulmonary capillary leakage in humanized mice, sarpogrelate was employed (84). Administration of this drug to antibody-mediated TRALI in humanized mice abolished the exacerbation of lung edema (84). The significant effect of sarpogrelate on TRALI suggested that targeting activated platelet-mediated serotonin secretion may represent a potential TRALI therapy. Alternatively, tirofiban—a platelet receptor antagonist—possessed potential for preventing murine 34-1-2S-mediated TRALI *via* suppressing platelet activation and targeting pulmonary coagulation-fibrinolytic disorders (134).

##### 3.2.3.7 Targeting Tregs/IL-10

Tregs are a subpopulation of CD4^+^ T cells that maintain tissue tolerance *via* secretion of anti-inflammatory cytokines (e.g., IL-10) (154, 155). Treg-based therapies have been proven promising for autoimmune diseases and allograft rejection (155, 156). In particular, IL-2 is a trophic cytokine required for the expansion of effector cells as well as Tregs (135), and the IL-2/anti-IL-2 antibody complex (IL-2c) induces vigorous T-cell proliferation *in vivo* (135). In a murine antibody-mediated TRALI model, administration of IL-2/IL-2c rapidly expanded the Treg population, thereby increasing the level of IL-10, which enabled mice to recover from lung injury (157). Given that IL-10 possesses potent anti-inflammatory and tissue regenerative capabilities, IL-10 administration prophylactically protected and therapeutically rescued against TRALI *in vivo* (13, 32, 158). These findings indicate that induction of Treg IL-10 production, or direct IL-10 treatment represent promising therapeutic strategies for TRALI.

## 4 Conclusions

Although TRALI is a leading cause of transfusion-associated death, specific treatments are still lacking. Thus, understanding its pathogenesis is key to establishing effective disease management strategies. Recently, significant progress has been made in defining TRALI pathophysiology. In particular, based on the two-hit theory, we innovatively define a three-step pathogenesis model for TRALI that is composed of the priming step, pulmonary reaction, and effector phase. As depicted in this model, the patient risk factors, in combination with detrimental factors within the blood transfusion components, trigger a dynamic spectrum of clinical manifestations based on complex host responses. These responses involve immune cells, including PMNs, platelets, Tregs, monocytes, macrophages; non-immune cells, such as ECs; and effector molecules, such as NETs, ROS, and coagulation-fibrinolytic disorders. In fact, the three-step pathogenesis model especially in pulmonary reaction and effector phase may be potentially overlapping.As such, application of a TRALI pathogenesis stage-specific management strategies may serve to improve disease progression. Hence, a better understanding of modifiable risk factors may help identify those patients most susceptible to TRALI onset. Following the introduction of strategies to exclude anti-leukocyte antibodies, BRMs, and associated pathogenic molecules from blood products, the incidence of TRALI in clinical studies and preclinical models has decreased rapidly. Additionally, a large number of *in vivo* drug experiments targeting PMNs, macrophages, platelets, Tregs, ECs, NETs, ROS, and coagulation-fibrinolytic disorders have effectively prevented the occurrence of lung edema. As these interventions have demonstrated efficacy in preventing TRALI occurrence in preclinical studies, their early implementation might benefit disease prognosis. One potential therapeutic agent is ascorbic acid, which has been proven beneficial for TRALI recovery in critical patients within a clinical trial. Further in-depth research is required to investigate effective drugs for targeted therapies to achieve better clinical outcomes.

## Author contributions

YY and ZL conceptualized the review. YY drafted the manuscript, created the figures, and constructed the tables. ZL edited the final version of manuscript. ZL obtained the funding and provided the financial resource. All authors contributed to the article and approved the submitted version.
